# Pain Generators in Calcaneal Malunion: Evaluation of a Case Series With Mixed Treatment

**DOI:** 10.7759/cureus.108714

**Published:** 2026-05-12

**Authors:** Akshat Gupta, Siddhartha Sharma, Sharad Prabhakar, Mandeep Dhillon

**Affiliations:** 1 Orthopedics, All India Institute of Medical Sciences, Rajkot, Rajkot, IND; 2 Orthopedics, Postgraduate Institute of Medical Education and Research, Chandigarh, Chandigarh, IND

**Keywords:** calcaneal malunion, displaced intra-articular calcaneal fracture, heel pain, subtalar arthritis, treatment

## Abstract

Introduction

Chronic heel pain secondary to malunited fractures of the calcaneus can be quite disabling in nature. The etiology of pain depends upon the modality of primary treatment (i.e., operative or nonoperative), as well as the quality of articular reduction. While a number of probable causes have been identified, none of these pain generators have been adequately researched.

Methods

A total of 32 patients were prospectively evaluated. All had residual heel pain following calcaneal fracture and were divided into two groups (operated and non-operated) based on their primary treatment. The cause of heel pain was identified based on clinical examination findings as well as radiological results. Functional parameters used to objectify pain and disability were the Visual Analogue Scale and the American Orthopaedic Foot & Ankle Society hindfoot scores.

Results

There were 10 feet in the operated group and 22 in the non-operated group. The most common cause of residual heel pain in the operated group was pain due to prominent hardware. In sharp contrast, subtalar arthritis and peroneal tendon impingement secondary to calcaneal malunion were primarily seen in patients managed conservatively. Calcaneal fractures managed with anatomical reduction and fixation had better functional scores than those treated conservatively. Of the radiological parameters, Böhler angle and talocalcaneal height were significantly associated with the patients’ disability scores.

Conclusions

The traditional view among orthopedic surgeons that chronic pain in the sequelae of old intra-articular calcaneal fractures is secondary to subtalar arthritis only is incorrect. Patients undergoing fixation may have pain due to prominent hardware. All causes must be thoroughly assessed, as more than one may coexist.

## Introduction

Pain and disability are fairly common complications associated with long-standing fractures of the calcaneus [1]. This assumes even greater significance when we consider that the population most commonly affected is young males in their most productive years of life (age 20-45) [2].

Operatively treated displaced intraarticular calcaneal fractures (DIACFs), in which reduction is anatomical and reconstruction is adequately performed, tend to have better functional outcomes [3]. Nevertheless, surgical intervention is associated with its own set of challenges, which may lead to residual heel pain, such as sural nerve entrapment and pain due to prominent hardware [4,5].

Conservatively treated calcaneal fractures, on the other hand, frequently lead to malunion, and it is conventionally assumed that subtalar arthritis is the principal cause of residual heel pain in such cases [5,6]. However, this thinking has changed with a better understanding of the complex three-dimensional pathoanatomy of calcaneal malunion as well as hindfoot biomechanics, and other pain-generating factors have now been identified [5,7-14].

This issue of identifying pain generators after calcaneal fracture treatment has been inadequately researched. The present study was therefore designed to prospectively evaluate all old calcaneal fractures, irrespective of their primary management modality, and determine the various causes of residual heel pain in each case.

## Materials and methods

Study design

This was a prospective observational study conducted on patients attending the orthopedic outpatient clinic of a tertiary care institute in Northern India.

Study participants

Patients included in the study had isolated calcaneal fractures (more than 6 months old) and were between 14 and 65 years of age. Patients outside the specified age range, those with congenital abnormalities of the foot, and cases with sensorimotor loss due to associated head injury/paraplegia were excluded from the study. Diabetic feet and pathological fractures of the calcaneus were also excluded.

**Figure 1:**
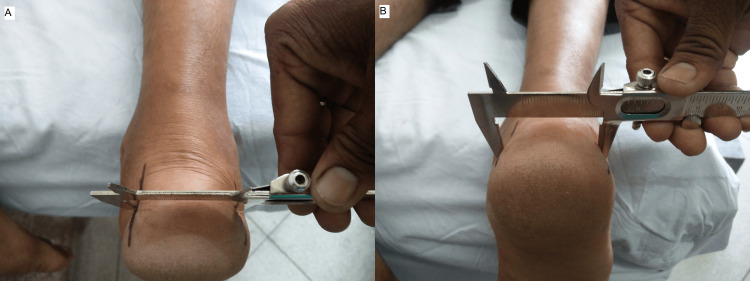
Clinical picture depicting measurement of heel width with the patient in the prone position

Patients were first clinically assessed after taking a detailed history, followed by physical examination, where special focus was placed on palpating individual surfaces of the calcaneus. Any varus or valgus deformity of the hindfoot was also documented. Subtalar and ankle range of motion was assessed, and heel width was measured (Figure 1A, 1B).

Based on clinical parameters, the severity of pain and disability was quantified using the Visual Analogue Scale (VAS) and the American Orthopaedic Foot & Ankle Society (AOFAS) hindfoot scores [15].

**Figure 2:**
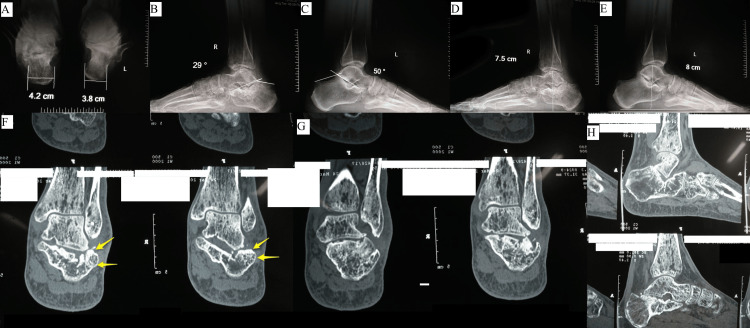
(A-E) Lateral and Harris axial views of the injured and normal calcaneus showing loss of TCH and BA and widening of the heel. (F, G) Coronal CT cuts depicting lateral wall blowout and subfibular impingement (yellow arrows). (H) Sagittal CT sections demonstrating arthritic changes in the subtalar joint BA, Böhler’s angle; TCH, talocalcaneal height

Imaging included standard AP, lateral, and oblique views of the foot. The lateral view was used to assess loss of talocalcaneal height (TCH) and Böhler’s angle (BA). A Harris axial view of the heel, along with CT of the calcaneus, was also obtained (Figure 2A-2E). These were used to measure calcaneal width (W) and quantify the extent of lateral wall blowout. The CT image sequences (Figure 2F-2H) were also used to grade the severity of calcaneal malunion as per the classification laid down by Stephens and Sanders [16].

After a thorough clinico-radiological assessment, the causes of residual heel pain were identified in all patients. Management involved an initial trial of analgesics, footwear modification, and activity limitation. Non-responders were considered for surgical management. Patients with painful hardware underwent removal of the affected implant. Follow-up for surgical cases was at six weeks, three months, and thereafter every six months until the latest follow-up. At each visit, patients were evaluated using VAS and AOFAS scores as well as the aforementioned radiological parameters.

Ethical considerations

All patients were enrolled in the study after providing written informed consent. Approval was obtained from the institutional ethics committee.

Statistical analysis

Continuous data were expressed as mean ± SD. Comparison of function and pain scores between study groups was performed using the nonparametric Mann-Whitney U test (IBM SPSS Statistics for Windows, version 29.0 (released 2022; IBM Corp., Armonk, NY, USA)). Correlation was assessed using Pearson’s correlation coefficient. Statistical significance was set at p < 0.05 with 95% CIs.

## Results

**Figure 3:**
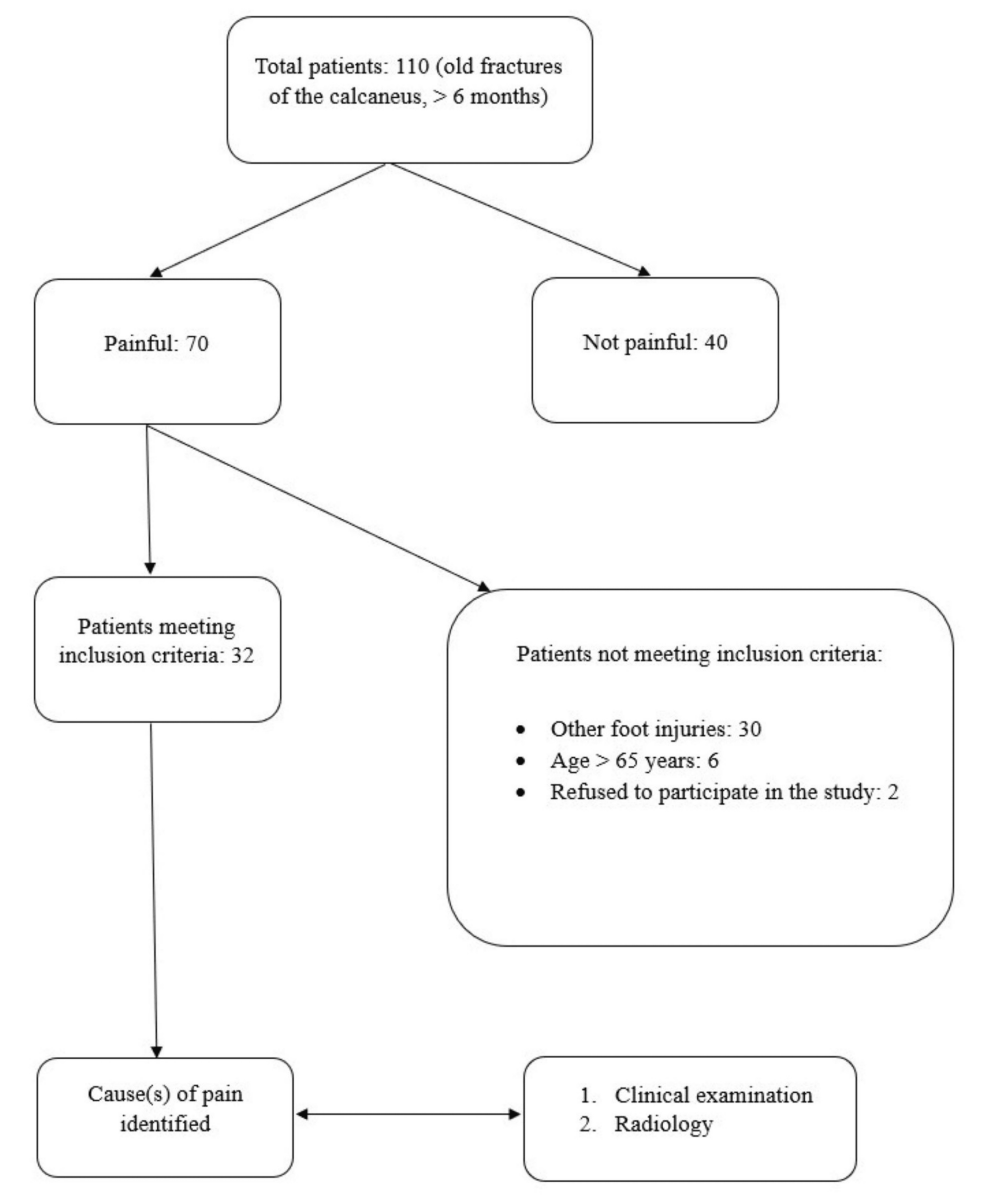
Flowchart summarizing the patient selection process and study methodology

Out of a total of 110 old calcaneal fractures who presented to us, 70 patients had a painful heel and were subsequently evaluated for this study. Thirty of these 70 were excluded on account of other foot injuries, and six did not meet the predetermined age criteria of the study; two patients refused to give consent. The study cohort thus comprised 32 cases who were then evaluated (Figure 3).

The average age of the patients was 38 ± 9.6 years, with 27 male subjects and five females. The most common mechanism of trauma was fall (29/32 cases, 91%), followed by road traffic accident (3/32 cases, 9%). Four patients had bilateral fractures of the calcaneus. However, in all four, only one side was painful. Hence, the number of painful heels evaluated did not change (n = 32).

The average follow-up of conservatively and operatively treated fractures was 5.5 ± 1.1 and 5.4 ± 0.8 months, respectively. Six patients had a history of chronic smoking.

Twenty-eight of 32 were closed fractures, and four were open injuries (three: open grade IIIA and one: open grade IIIB). Ten patients had an initial operative intervention (six: open reduction and internal fixation (ORIF); two: minimally invasive surgery (MIS); and two cases of open calcaneal fracture: debridement and K-wire fixation). The remaining 22 patients had been managed conservatively with compression bandage application and splinting of the affected limb. These included two open fractures of the calcaneus that underwent only debridement of the fracture wound.

The average duration from primary injury to the time of presentation with residual pain was 1.9 ± 2.6 years. Functional evaluation revealed average AOFAS and VAS scores of 65.8 ± 12.9 and 4.9 ± 1.9, respectively. Radiographic measurements, on the other hand, were as follows: (i) BA: 14.9 ± 6.7 degrees; (ii) W: 3.9 ± 0.5 cm; and (iii) TCH: 6.4 ± 0.8 cm.

**Figure 4:**
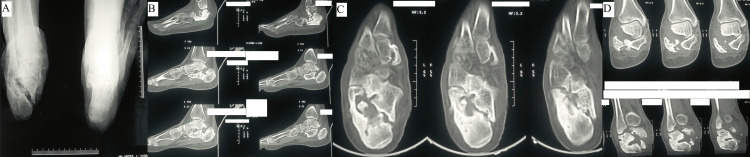
Radiographs and CT scan of the patient with associated nonunion of the calcaneus

Twenty-five out of 32 cases had features of calcaneal malunion (78.1%). This included six cases of type 1, 14 cases of type 2, and five patients of type 3 malunion. One patient with Sanders type 2 malunion had associated nonunion of the calcaneus (Figure 4A-4D).

Clinico-radiological assessment revealed a single cause of pain in 18 patients, while in the remaining 14 subjects, two or three causative factors were identified.

**Figure 5:**
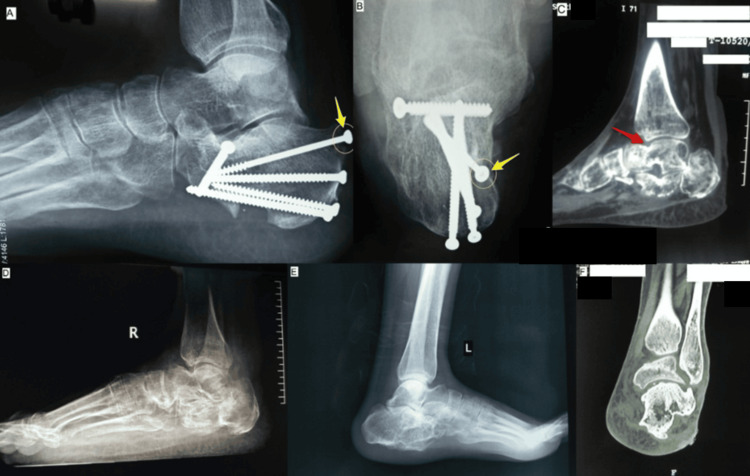
(A, B) X-rays showing the calcaneus fracture treated previously with percutaneous screws - patient complained of pain due to a prominent screw head (yellow circles). (C, D) Dorsiflexed ankle (red arrow) causing anterior tibiotalar impingement. (E, F) Plantar exostoses leading to heel pad pain in the patient

Out of 10 patients who were initially operated on, six (five in ORIF and one in MIS) had pain due to prominent hardware (Figure 5A, 5B). Two of four cases that underwent debridement with K-wire fixation had subtalar arthritis. There were no wound-related complications in any of the cases; one case had a burning sensation in the foot in addition to hardware-related pain. He was diagnosed with a case of iatrogenic sural nerve injury. In patients managed conservatively, the predominant pain generators were peroneal tendon impingement and subtalar arthritis secondary to calcaneal malunion (16/22 cases each). One instance of anterior tibiotalar impingement was observed in a patient with an open grade IIIB calcaneus fracture (Figure 5C, 5D), while another patient had a painful heel secondary to plantar exostoses (Figure 5E, 5F).

In all instances, DIACFs primarily managed with anatomical reduction and fixation, either via ORIF or MIS, had better functional and pain scores than those treated conservatively, even if the difference was not statistically significant (p > 0.05). Radiological parameters, however, were significantly better in the operative group (p < 0.05) (Table 1).

**Table 1:** Comparison of operatively treated patients (ORIF/MIS) with those managed conservatively in terms of functional and radiological parameters AOFAS, American Orthopaedic Foot & Ankle Society; BA, Böhler angle; MIS, minimally invasive surgery; ORIF, open reduction and internal fixation; TCH, talocalcaneal height; VAS, Visual Analogue Scale; W, calcaneal width

Parameter	Group A (operated)	Group B (non-operated)
Total feet	10	22
Cause of pain		
Painful hardware	6/10	Nil
Subtalar arthritis	4/10	16/22
Peroneal impingement	2/10	16/22
Sural neuritis	1/10	Nil
Anterior tibiotalar impingement	Nil	1/22
Plantar exostoses	Nil	1/22
AOFAS (Mann-Whitney U test, p = 0.115)	70.5 ± 12.7	63.6 ± 12.7
VAS (Mann-Whitney U test, p = 0.167)	4.2 ± 2	5.2 ± 1.9
BA (degrees) (Mann-Whitney U test, p = 0.048)	18.1 ± 6.6	13.4 ± 6.4
W (cm) (Mann-Whitney U test, p = 0.014)	3.6 ± 0.4	4.0 ± 0.4
TCH (cm) (Mann-Whitney U test, p = 0.048)	6.8 ± 0.8	6.2 ± 0.7

Correlation of patients’ pain and disability scores with radiological parameters revealed a significant association of AOFAS and VAS scores with corresponding BA and TCH values (p < 0.05).

## Discussion

Fractures of the calcaneus account for approximately 2% of all bony injuries and 60-70% of tarsal fractures [5,17,18]. Chronic heel pain is a common sequela of these injuries and is reported in about 38-80% of cases [19,20]. The etiology of pain is often multifactorial, and multiple pain generators may be responsible for pain in a single patient [6]. In order to identify them, it is important to understand the pathological anatomy of calcaneal malunion and hindfoot biomechanics.

The primary fracture line in most DIACFs separates the calcaneal tuberosity from the sustentaculum tali, while the secondary line travels in the sagittal plane along the length of the bone [5]. As a result, while the sustentaculum remains attached to the talus via the interosseous ligaments (the so-called “constant fragment”), the tuberosity is displaced laterally. This gives rise to the following deformities characteristic of calcaneal malunion: (i) loss of calcaneal height; (ii) heel widening with lateral wall blowout; and (iii) varus/valgus deformity of the heel [5,21-23].

There are six surfaces of the calcaneus, and causes of heel pain have accordingly been described for each [5,21,24]:

Lateral surface

There are two major causes of lateral-sided heel pain in malunited calcaneal fractures: peroneal tendon impingement and subtalar arthritis. The former is typically associated with loss of active foot eversion with occasional swelling [5,8,18,25]. Subtalar arthrosis, on the other hand, has a more delayed presentation and is more likely to be seen in DIACFs managed conservatively [3,14,26]. Similar findings were also observed in our study.

In addition to the above two, a secondary extension of the fracture line into the calcaneocuboid joint can sometimes be seen in 48% of cases [27]. Such patients tend to have more anteriorly localized lateral heel pain.

A number of studies have also reported instances of implant-related pain following ORIF or MIS for DIACFs. These may be due to prominent screw heads [6] or irritation neuritis of the sural nerve by the locking plate [5,9]. In our analysis, six patients had hardware-related pain, of whom five were secondary to ORIF. Of the latter, four had been operated on at our institute and one outside. The patient operated elsewhere also had clinical features suggestive of sural nerve injury.

Plantar surface

According to Lindsay and Dewar [11], plantar heel pain can be seen in up to 26-38% of patients after foot injury. In a study by Silver et al. [28] on ultrasound measurements of heel pad thickness in patients with calcaneal fracture, a significant increase in thickness was demonstrated on the affected side. Cohen has theorized that injury to the heel pad can be either due to direct trauma or secondary to loss of TCH. The latter causes a drop in the pitch angle, which in turn leads to lengthening of the medial arch of the foot. This results in excessive stretching of the plantar fascia, thereby leading to pain [22].

Banerjee et al. [5] have described bony spurs/exostoses as another cause of pain in patients with calcaneal malunion. These are usually asymptomatic and detected only on imaging. This was in contrast to our study group, where one patient with plantar exostoses had pain localized to the base of the heel.

Medial surface

A few authors have reported medial-sided heel pain following injuries to the calcaneus. These are mostly due to tarsal tunnel syndrome or flexor hallucis longus tendinopathy secondary to fractures of the sustentaculum tali [12,29]. Neither of these was noted in our study population.

Anterior and dorsal surface

Lindsay and Dewar [11] reported a 22-35% occurrence of anteriorly/dorsally located pain in patients with malunited fractures of the calcaneus. The pain is usually reproduced on passive dorsiflexion of the ankle. We noted one such case of anterior tibiotalar impingement in a 56-year-old man who was also suffering from subtalar arthritis and peroneal impingement.

Posterior surface

Lui [13] has shown that in some malunited DIACFs, the bony spike just behind the depressed posterior facet of the subtalar joint can lead to posterior ankle impingement, causing pain during plantarflexion.

Complex regional pain syndrome (CRPS)

According to Haugsdal et al. [9], neuropathic pain can result not only from sural nerve irritation but also from idiopathic mechanisms such as CRPS. In such cases, patients may present with a host of symptoms, such as pain out of proportion to trauma and trophic skin changes.

Management of malunited DIACFs requires adequate evaluation to correctly identify the source of pain. Toward that end, Myerson and Quill have found fluoroscopy-guided selective analgesic injections of the nerves and joints to be particularly helpful [30]. In addition, plain radiographs of the foot as well as CT scans are vital tools that aid in determining the extent and location of arthrosis. Coronal cuts, in particular, are useful in quantifying the extent of calcaneal malunion, which in turn determines the nature of surgery to be performed.

Limitations

The main drawback of our study was the limited sample size, which may limit the applicability of the results to the broader population. Another drawback was the lack of matching between the two study cohorts with respect to initial injury severity.

## Conclusions

The cause of residual heel pain in malunited calcaneal fractures is often multifactorial and can vary depending on the primary mode of management. Subtalar arthritis and peroneal tendon impingement secondary to lateral wall exostoses represent the most common etiology in conservatively managed cases. Hardware-related problems, such as pain and sural nerve entrapment, on the other hand, are more likely to occur in patients undergoing surgery. All cases require thorough evaluation, combining clinical findings with radiological inputs, to make an accurate diagnosis. In the future, prospective studies with larger sample sizes can be planned to draw more definitive conclusions.
